# Bone marrow monocytes and macrophages from mice lacking βENaC and ASIC2 have a reduced chemotactic migration response and polarization

**DOI:** 10.14814/phy2.16139

**Published:** 2024-07-17

**Authors:** Robert Wasson, Adam B. Fleming, Je’la McLin, Emily Hildebrandt, Heather A. Drummond

**Affiliations:** ^1^ School of Medicine University of Mississippi Medical Center Jackson Mississippi USA; ^2^ Mississippi INBRE Research Scholar Mississippi State University Starkville Mississippi USA; ^3^ Department of Physiology and Biophysics University of Mississippi Medical Center Jackson Mississippi USA

**Keywords:** degenerin, interferon gamma, myeloid, TNFalpha

## Abstract

The monocyte–macrophage system plays an important role in phagocytosis of pathogens and cellular debris following infection or tissue injury in several pathophysiological conditions. We examined ENaC/ASIC subunit transcript expression and the importance of select subunits in migration of bone marrow derived monocytes (freshly isolated) and macrophages (monocytes differentiated in culture). We also examined the effect of select subunit deletion on macrophage phenotype. BM monocytes were harvested from the femurs of male and female WT and KO mice (6–12 weeks of age). Our results show that α, β, γENaC, and ASIC1‐5 transcripts are expressed in BM macrophages and monocytes to varying degrees. At least αENaC, βENaC, and ASIC2 subunits contribute to chemotactic migration responses in BM monocyte–macrophages. Polarization markers (CD86, soluble TNFα) in BM macrophages from mice lacking ASIC2a plus βENaC were shifted towards the M1 phenotype. Furthermore, select M1 phenotypic markers were recovered with rescue of βENaC or ASIC2. Taken together, these data suggest that βENaC and ASIC2 play an important role in BM macrophage migration and loss of βENaC and/or ASIC2 partially polarizes macrophages to the M1 phenotype. Thus, targeting ENaC/ASIC expression in BM macrophages may regulate their ability to migrate to sites of injury.

## 1 INTRODUCTION

The monocyte–macrophage system is the first responder of the immune system to tissue injury and infection. Monocytes and macrophages are phenotypically mutable. Monocytes can differentiate into macrophages and macrophages can polarize into a proinflammatory (M1) macrophage phenotype by Interferon Gamma (IFNγ) and Tumor Necrosis Factor Alpha (TNFα), among other cytokines, to promote inflammation and phagocytose damaged tissue. Macrophages can also be polarized into anti‐inflammatory (M2) phenotype by select cytokines including Interleukin‐4, or ‐10, leading to inflammation resolution and tissue repair (Murray, 2017).

Circulating monocytes are attracted to and accumulate near the site of injury. Monocytes then invade the tissue and migrate towards the site of injury. Migration is a complex process: the circulating monocyte–macrophages must (1) first sense the chemo‐attractants released from the injury site and accumulate, (2) then release enzymes to disrupt the extracellular matrix and cell‐to‐cell contacts to create a pathway for invasion, and (3) continually disrupt then reform contacts between cell surface molecules and the extracellular environment and reorganize the cytoskeleton as it migrates. As macrophages reach their target, they become polarized, release cytokines and chemokines to attract additional immune cells, then phagocytose damaged tissue and initiate repair processes (Italiani & Boraschi, 2014; Miskolci et al., 2021; Murray, 2017; Orekhov et al., 2019; Santisteban & Iadecola, 2018; Vogel et al., 2014).

To migrate normally, macrophages must be able to interact with their extracellular environment by sensing chemical and mechanical cues. Degenerin ion channels are candidates to integrate these cues in macrophages. Degenerin proteins are known to operate as chemosensors and mechanosensors in a variety of cell types. Acid Sensing Ion Channel (ASIC) subunits form extracellular H^+^ gated cation channels in neurons and smooth muscle (Bianchi & Driscoll, 2002; Drummond, 2021; Kellenberger & Schild, 2002; Syntichaki & Tavernarakis, 2004). Epithelial Na^+^ Channel (ENaC) and select ASIC subunits can act as mechanosensors in neurons, epithelial, endothelial, and smooth muscle cells (Bianchi & Driscoll, 2002; Drummond, 2021; Kellenberger & Schild, 2002; Knoepp et al., 2020; Satlin et al., 2001; Syntichaki & Tavernarakis, 2004). Additionally, several studies suggest certain degenerins are critical to neuritogenesis (Drummond et al., 2006; Fuller et al., 2023; Tao et al., 2022; Zha et al., 2009), a process that is guided by integration of chemical and mechanical cues, similar to migration. Several empirical studies suggest that degenerin family members are expressed in cells of myeloid origin including peripheral blood mononuclear cells, monocytes, splenic and bone marrow derived dendritic cells, and monocyte–macrophage cell line RAW cells (Barbaro et al., 2017; Ertuglu et al., 2024; Nemeth et al., 2022; Ni et al., 2018; Reus‐Chavarria et al., 2019). However, which degenerin subunits are expressed in bone marrow derived monocytes and macrophages has not been studied.

A previous study from our laboratory in the RAW monocyte–macrophage cell line suggests that ENaC is required for normal chemotactic migration (Nemeth et al., 2022). Furthermore, polarization to M1 phenotype using IFNγ ± TNFα inhibits of αENaC expression and motility. Surprisingly, loss of ENaC activity induced a subtle morphological and phenotypical change consistent with an M1 macrophage raising the possibility that ENaC inhibition may be a mechanism of polarization. However, whether ENaC or other ASIC subunits play a critical role in bone marrow derived monocyte and macrophage migration and polarization has not been addressed.

In the current investigation, we examined ENaC/ASIC subunit transcript expression and the importance of select subunits in migration of freshly isolated bone marrow derived monocytes and monocytes differentiated into macrophages in culture. We also examined the effect of select subunit deletion and rescue on macrophage polarization status.

## 2 METHODS

### 2.1 Animals

Male and female wildtype and genetically modified mice (ASIC2^−/−^, βENaC^m/m^, and ASIC2^−/−^/βENaC^m/m^) were maintained as homozygous mating pairs on C57BL/6 background. Global ASIC2 knockout mice are available through Jackson Labs (RRID: IMSR_JAX:013126). Hypomorphic βENaC (βENaC^m/m^) mice are available through the European Mutant Mouse Archive (RRID: IMSR_EM:04574). Global βENaC knockout mice are not available as mice die shortly after birth (McDonald et al., 1999). Our laboratory previously crossed ASIC2^−/−^ onto βENaC^m/m^ mice to generate ASIC2^−/−^/βENaC^m/m^ animals (Kleyman et al., 2009). Animals were housed under 12 h light–dark cycles, provided normal chow (0.3% Na^+^, Teklad Cat # T.8604) and water ad libitum. All mice were used between 6 and 12 weeks of age. Offspring were genotyped at 3 weeks of age using DNA (DirectTail PCR, Viagen Cat #102‐T) isolated from ear punch or tail snip samples, then reconfirmed following tissue isolation using liver samples. All protocols were reviewed and approved by the Institutional Animal Care and Use Committee of the University of Mississippi Medical Center.

### 2.2 Bone marrow monocyte–macrophage isolation and culture

Mice were anesthetized with isoflurane (NDC Cat # 66974–017‐25) until respiration ceased, followed by cervical dislocation, then the femurs and tibias were quickly removed and placed on ice. The marrow was flushed from the bones with sterile Physiological Buffered Saline (PBS, Fisher Science, Cat # BP399) with EDTA (Invitrogen Cat # 15575–038) and fetal bovine serum (FBS, Corning Cat # 35‐010‐CV) and processed following the manufacturer's protocol. Bone marrow monocytes were isolated using Miltenyi Monocyte Isolation Kit (Catalog #130–100‐629) and Miltenyi magnetic LS columns (Cat # 130–042‐401), a protocol in which non‐monocyte populations are depleted from the bone marrow cell preparation. Cells were either used immediately for migration experiments (BM derived monocytes) or placed in culture and differentiated to macrophages (BM derived macrophage). For differentiation to macrophages, 1 × 10^6^ freshly isolated monocytes were plated on T75 flasks (Fisher #FB012937) in Opti‐MEM (Gibco, Cat # 31985–070) plus 10% heat inactivated FBS (30 min at 50°C), 1% penicillin–streptomycin (Corning, Cat # 30‐002‐CI), and 20 ng/mL macrophage colony‐stimulating factor 1 (CSF1, R&D Systems, Cat # 486‐ML‐50). Macrophages were expanded in culture by using trypsin (4 min at 37C; Corning Cat # 25‐053‐CI) following by gentle scraping then replating on T75 flasks at 1:3 ratio. Macrophages were cryopreserved (Recovery Freezing Medium, Gibco, Cat # 12648–010) at 1 × 10^6^ cells/mL then stored in liquid nitrogen for later use. Macrophages were studied at passages 6–18. Cells were continuously monitored for proliferation and appropriate morphology. Macrophages at similar passages were used for all comparisons within a given experiment.

### 2.3 RNA extraction

Macrophages on T75 flasks were rinsed with sterile DPBS, scraped into 6 mL ice‐cold DPBS (Gibco, Cat # 140‐40133), transferred to collection tubes, and centrifuged at 3000 rpm for 20 min, at 4°C. DPBS was removed and cell pellets stored at −80°C. To extract RNA from monocytes, bone marrow samples were pooled from four animals, then stored at −80°C in Trizol (Zymo Research, Cat #R2050‐1). Harvested cells were resuspended in 300 μL of cold (~4°C) Trizol, and total RNA was isolated using Quick‐RNA Microprep Kit (Zymo Research, Cat #R1050). RNA samples were stored at −80°C. RNA was reverse transcribed using the iScript Advanced cDNA Synthesis Kit (Bio‐Rad, Cat # 1725038). Reactions were incubated at 25°C for 5 min, 46°C for 20 min, and 95°C for 1 min.

### 2.4 Quantitative polymerase chain reaction (qPCR)

qPCR was used to determine (1) mouse α, β, and γENaC, ASIC1–5 gene expression in bone marrow derived macrophages and freshly isolated monocytes and (2) impact of βENaC and ASIC2 loss on expression of macrophage polarization markers (CD68, CD86, CD163, and CD206). Glyceraldehyde 3‐phosphate dehydrogenase (Gapdh) gene expression was used as an internal control and to normalize gene expression. TaqMan® primer pair‐probe (FAM‐MGB) sets were obtained from Applied Biosciences (see Tables 1 and 2). Primer probe sets were tested in 10, 100, and 1000 ng RNA equivalent samples from lung, liver, kidney, and brain samples. Robust PCR products at the expected size were found in the expected tissues. Results for ENaC targets are shown in Nemeth et al. Results for ASIC targets are not shown. Several ASIC2 and ASIC1 primer‐probe sets were evaluated. PCR reactions, using BioRad Supermix for Probes (No dUTP, Cat #1863024), were incubated at 95°C for 10 min, followed by 45 cycles of 94°C for 30 s, and 60°C for 1 min, using a ViiA 7 Real‐Time PCR System (Applied Biosciences) with MicroAmp Fast Optical 96‐Well Reaction Plates [R&D Systems, Inc. (Cat # 4346906)]. For all targets in macrophages, we used 100 ng RNA equivalent, except ASIC1 where we used 1000 ng. For samples in freshly isolated monocytes, we used 75 ng RNA equivalent from column purified monocytes. PCR reactions for ENaC, ASIC, and Gapdh were separated on 3%–4% agarose (Fisher Science, Cat # BP1356) gels to ensure that an amplicon of appropriate size was present. GeneRuler Low Range DNA Ladder (Thermo Scientific, Cat # SM0241) was used to estimate size. All samples were run in triplicate.

**TABLE 1:** TaqMan Gene Expression Assay Primer‐Probe Pairs for mouse ENaC Subunits.

Gene	Protein	RefSeq ID	Assay name	Exon boundary	Amplicon length (bp)
SCNN1a	αENaC	NM_011324.2	Mm00803386_m1 ^b^	2‐3	69
SCNN1b	βENaC	NM_001272023.1	Mm00441215_m1 ^b^	4‐5	83
SCNN1g	γENaC	NM_11326.2	Mm00441228_m1 ^b^	6‐7	63
ACCN2	ASIC1	NM_009597.1	Mm01305997_m1^b^	3–4	65
			Mm01306004_g1	8–9	62^a^
		NM_001289791.1	Mm01306001_g1	5‐6	72^a^
ACCN1	ASIC2	NM_001034013.2	Mm01304218_m1^b^	8–9	99
			Mm00475691_m1	5‐6	79^a^
			Mm01304217_m1	7‐8	149^a^
	ASIC2b	NM_007384.3	Mm00475687_m1	1–2	105
ACCN3	ASIC3	NM_183000.2	Mm00805460_m1 ^b^	1–2	82
ACCN4	ASIC4	NM_183022.3	Mm00805551_m1 ^b^	1–2	112
ACCN5	ASIC5	NM_021370.2	Mm00517541_m1 ^b^	8–9	130
Gapdh	GAPDH	NM_008084.2	Mm99999915_g1^b^	3	107

*Note*: ASIC1 Mm01306004 did not detect a band at predicted size. ASIC1 Mm01306001 did not detect band at predicted size. ASIC2 Mm00475691 detected 2 bands, 1 at predicted size. ASIC2 Mm01304217 did not detect band at predicted size. ASIC2b was detected in 1 out of 4 different macrophage samples, at predicted size.

^a^ Multiple Primer‐Probe pairs were tested for ACCN2, ACCN1.

^b^ The blue highlighted “Assay Name” was used in the current study.

**TABLE 2:** TaqMan Gene Expression Assay Primer‐Probe Pairs for Macrophage (M1/M2) Polarization.

Gene	Assay name	Cell type marker
CD68	Mm03047343_m1	Monocyte origin
CD86	Mm01344638_m1	M1 polarization
CD163^a^	Mm00474091_m1	M2 polarization
CD206/MRC1/Mannose Receptor	Mm01329362_m1	M2 polarization
PPARγ^a^	Mm00440940_m1	M2 polarization

^a^ C_T_ not detected in any samples up to 45 cycles.

PCR data were analyzed using QuantStudio™ Real‐Time PCR Software v1.6.1. (Applied Biosystems). All amplification curves were examined for logarithmic amplification, baseline stability, noise spikes, and outliers. Trials where the Cycle Threshold (C_Th_) values for Gapdh varied greater than 1 cycle among groups were excluded from analysis. C_Th_ values were determined by QuantStudio software using the “relative analysis” setting. To quantify fold expression, we used the delta delta C_Th_ method, where fold expression = 2^−∆∆CT^.

### 2.5 Stable transfection

To inhibit expression of αENaC and βENaC in macrophages, wildtype macrophages were plated on a 6 well plate and transfected at 50% confluency with 4 μg of dominant‐negative constructs (1:0.7:0.5, DNA: Lipofectamine 3000: Enhancer: DNA; Invitrogen Cat # L3000001) in 300 μL of Opti‐MEM. The constructs were formed by the first 112 amino acids of mouse αENaC or the first 41 amino acids of βENaC fused to the C‐terminus of Enhanced Green Fluorescence Protein (EGFP_αENaC_W112X_ or EGFP_βENaC_I41X_). EGFP alone was used as a control. Similar N‐terminal truncations of ENaC constructs downregulate endogenous subunit expression (Grifoni et al., 2006; Jernigan & Drummond, 2006; Montano et al., 2009; Nemeth et al., 2022; Pochynyuk et al., 2008).

### 2.6 Western blot

We used standard Western blotting techniques to assess αENaC and βENaC expression in freshly isolated bone marrow monocytes. For these experiments, monocytes from two male and two female mice were pooled into a single sample. Cells were lysed in 100 μL of KBO buffer (25 mM Na_3_PO_4_, 300 mM NaCl, 0.5% Triton X‐100, and 20 mM octyl glucoside, pH 7.4), then centrifuged at 10,000 rpm for 30 min at 4°C and separated into soluble and insoluble fractions. The soluble fraction was quantified using Biorad Detergent Compatible Bradford Assay Kit (Cat #5000111) and 20 μg was assayed. Protein samples were heated at 100°C for 10 min in 5X Sample Buffer plus DTT (Pierce, Cat #39000) then separated on 7.5% SDS‐PAGE (Biorad, Cat # 5671024) and transferred to nitrocellulose (Biorad, Cat #1620112). Blots were blocked using Odyssey Blocking Buffer (LiCor, Cat #927‐70001), incubated with rabbit anti αENaC (Stressmark) or rabbit anti‐βENaC (Drummond Lab, 1:1000) and incubated overnight, followed by mouse anti‐βActin for 1 h. Primary antibody labeling was visualized using Donkey anti‐Rabbit IR680RD and Donkey anti‐Mouse IR800CW with an Odyssey Infrared Scanner.

To rescue the expression of βENaC or ASIC2 in macrophages from ASIC2^−/−^/βENaC^m/m^ animals, we transfected macrophages with full length mouse βENaC or ASIC2a fused to the C‐terminus of EGFP or ECFP, respectively (EGFP_βENaC and ECFP_ASIC2a). EGFP alone was used as a control cell line. To enrich the population of cells expressing the truncated or full‐length constructs, cells were cultured in the presence of G418 (Roche, Cat # 04727878001) at 1:100 for ~5 passages to generate a stable expressing cell line. EGFP/ECFP expression was confirmed using fluorescence localization. Fluorescence was marginally detected in transfected cells grown in the presence of G418. Un‐transfected control cells died within 3 days of exposure.

### 2.7 Secreted TNFα

To detect secretion of TNFα in the media from wildtype control, knockout, and rescue‐transfected macrophages, we assayed soluble TNFα using an ELISA assay (Biolegend, Cat # 430907) through our Analytic and Assay Core which uses standardized approaches. For these experiments, 5 × 10^5^ cells in 2 mL were seeded per well on 6 well plates and grown for additional 72 h. Media was collected and stored at −80 until analysis. A standard curve using 15, 30, 60, 125, 250, 500, and 1000 pg/mL TNFα was used. TGFβ (Novos, Cat # NBP1‐92671), IL1b (R&D Systems, Cat #MHSLB00), IL6 (R&D Systems, Cat #M6000B), IL10 (R&D Systems, Cat #M1000B), IL17 (R&D Systems, Cat #M1700), and iNOS (Novus Bio Cat #NBP2‐80256) were undetectable. Samples for wildtype control versus KO and KO versus rescue with βENaC/ASIC2 were collected in separate experiments, but assayed side‐by‐side. Macrophage samples from two different wells derived from three different animals per group were used.

### 2.8 Immunolabeling and confocal imaging

For immunolabeling, cells were plated on 8 well chambered cover‐glass slides. Near 70% confluency, samples were rinsed with DPBS, fixed in 4% paraformaldehyde for 10 min, then rinsed in DPBS. Samples were treated with the Fc blocking reagent mouse (1:100, Miltenyi, Cat #130‐092‐575), blocked in 5% donkey serum (Jackson Immuno Research, Cat #017‐000‐121) for 1 h, then incubated with primary antibodies overnight at 4C. Samples were rinsed then incubated with donkey anti‐rabbit Alexa 546 secondary antibodies in 5% donkey serum, rinsed, cover‐slipped, and dried. Primary and secondary antibodies, titers, catalog numbers, and RRIDs are provided in Table 3. Samples were imaged using a Leica TCS SP8 confocal microscope, sequential channel scanning at 1024 × 1024 pixels, using a 63X objective under identical conditions for all samples within an experiment. To quantitate fluorescence, regions of interest were drawn around cell borders determined by overlaid signal in 25–30 cells from 2 to 3 images/group by a naive, blinded operator. Fluorescence was normalized to cell area. Images were prepared for presentation in Photoshop and represent original scans. Modifications, where used, were applied to all images within an experiment. Control samples without primary antibody had no signal.

**TABLE 3:** Primary antibodies, species, titer, and source.

Primary antibodies			
Antibody target	Host species	Titer	Commercial source/RRID or citation
αENaC	Rabbit	1:100	Alomone (Cat #ASC‐030, RRID AB_10917439), Residues 173‐185 of Rat αENaC (100% identity with mouse αENaC)
αENaC	Rabbit	1:1000	Stressmarq (Cat #SPC‐403, RRID:AB_10640131).
βENaC_C‐term_	Rabbit	1:100	Drummond Laboratory (Jernigan et al)
ASIC2_C‐term_	Rabbit	1:100	Drummond Laboratory (Gannon et al)
CD86‐Viobright 515	Human	1:50	Miltenyi Biotech (Cat# 130‐122‐136, RRID AB_2819414)
CD206	Mouse	1:300	Proteintech (Cat #60143, RRID:AB_2144924)
Actin, beta	Mouse	1:2000	AbCam (Cat #Ab6276, RRID:AB_2223210)

Secondary antibodies						
Host species	Target species	Fluorophore	Titer	Source	Cat#	RRID
Donkey	Rabbit	Alexa 546	1:500	Thermo	A10040	AB_2534016
Donkey	Rabbit	IRdye680RD	1:10,000	LiCor	925‐68073	AB_2716687
Donkey	Mouse	Alexa 488	1:500	Thermo	A32766	AB_2762823
Donkey	Mouse	Alexa 546	1:500	Thermo	A10036	AB_11180613
Donkey	Mouse	IRdye800CW	1:10,000	LiCor	925‐332212	AB_2716622

### 2.9 In‐vitro chemotactic migration assay

Macrophages were serum starved overnight in 0.4% FBS prior to migration. Monocyte and macrophage cells were counted using trypan blue (Invitrogen, Cat #T10282) exclusion, then 100,000 live cells in 100 μL (Opti‐MEM, plus 0.4% heat inactivated FBS, and 1% P/S) were loaded in the upper chamber of 8 μm pore Transwell inserts (Corning, Cat #3464) in a 12 well plate format. The lower chamber contained 500 μL Opti‐MEM, 10% standard FBS (non‐heat inactivated) plus 10 ng/mL CSF1. Preliminary experiments determined CSF1 in the lower chamber elicited a more robust response compared to no CSF1 or CSF1 in the upper chamber (data not shown). Inserts were incubated overnight. Insert upper surfaces were scraped with a cotton swab to remove unmigrated cells, gently rinsed in DPBS, fixed in 70% ice cold methanol for 10 min, stained with hematoxylin 10 min, then thoroughly rinsed under gently running water for 10 min. Cells on the bottom surface of the insert were counted on a Nikon Eclipse 200 inverted microscope using a 20× objective. Migration was quantified from seven fields of view/insert, from *n* = 3 inserts, for each group. Light microscopy images of representative fields of view were collected using a Nikon Eclipse 200 inverted microscope with 20× objective, CoolSnap Color camera, and Metamorph software.

### 2.10 Macrophage activation/proinflammatory cytokine treatment

Tumor Necrosis Factor alpha (TNFα, R&D Systems, Cat # 410‐MT‐010) and mouse Interferon gamma (IFNγ, R&D Systems, Cat # 485‐MT‐100) were used to induce macrophage polarization. Stocks were reconstituted with 0.1% bovine serum albumin (BSA, Sigma CAS# 9048‐46‐8) in sterile DPBS and stored in single‐use aliquots of 100 μg/mL at 20C. Macrophages were activated/polarized using IFNγ (10 ng/mL) during last 48 h plus TNFα (10 ng/mL, 0.4% FBS) during the last 24 h. To demonstrate morphologic changes in cells with cytokine treatment, light microscopy.

### 2.11 Statistical analysis

Data sets with similar variability were analyzed using 2‐tailed, independent *t*‐tests, one, two or three‐way ANOVA, using Prism 9 software, and presented as mean ± SEM. Data that did not pass a normal distribution test were analyzed using a Mann–Whitney Test (two groups) or Brown Forsyth/Welch Test (>2 groups). Post hoc comparisons to a control group use a Dunnett test, while comparisons among multiple groups used the Holm‐Sidak test. Bar graphs with individual data points are provided. Figure legends identify sample sizes and specific analyses applied. A value of *p* ≤ 0.05 is statistically significant. Result “trends” are also reported to avoid erroneous true/false conclusions based on bright‐line rules. Select *p* values are provided to demonstrate confidence.

## 3 RESULTS

### 3.1 Expression of ENaC and ASIC transcripts in male and female bone marrow derived macrophages and monocytes

**FIGURE 1:**
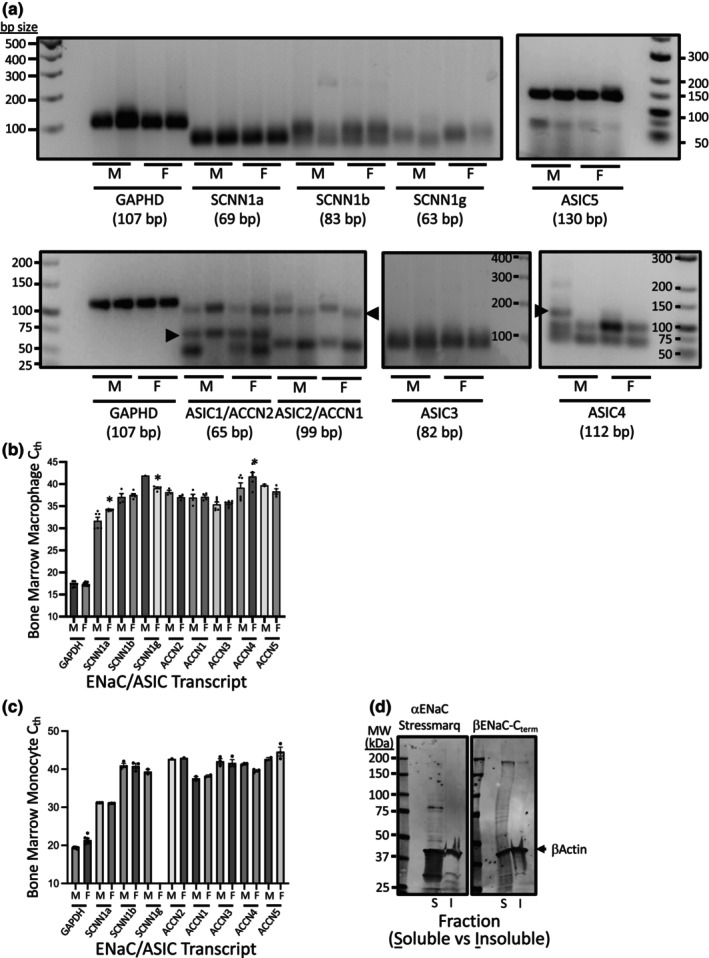
qPCR detection of α, β, γENaC, and ASIC1‐5 transcript expression in cultured bone marrow derived macrophages and freshly isolated monocytes from male and female mice. Macrophages were cultured in the presence of 20 ng/mL of CSF1. (a) PCR reactions from macrophages were separated on 3%–4% agarose gels to determine if amplicon of expected size was present (identified by arrowhead in samples with >1 product). 100 ng RNA template equivalent was used for all reactions except ASIC1, where 1000 ng was used. Three primer pair‐probe sets were tested on ASIC2 and ASIC1. The primer pair‐probe sets shown amplified a band at the expected size, in addition to 1–2 other bands. (b) Macrophage C_th_'s individual ENaC and ASIC transcripts from bone marrow derived macrophages 3 replicates in *n* = 2 trials. Thresholds at or near 35 cycles were consistently identified in all replicates for αENaC and ASIC3. Thresholds less than 39 cycles were identified for βENaC and ASIC2. γENaC amplified in only 1/6 and 4/6 replicates in male and female samples, respectively. (c) Bone marrow derived freshly isolated monocytes C_th_'s for individual ENaC and ASIC subunits using 75 ng RNA template equivalent. Samples from four animals were pooled. (d) Bone marrow derived freshly isolated monocyte Western blot detection of αENaC and βENaC. Samples were pooled from four animals. *Indicates statistical difference between male and female at *p* < 0.05 using an Uncorrected Fisher LSD Test.

Most ENaC and ASIC transcripts were detected in cultured macrophages derived from bone marrow monocytes and freshly isolated bone marrow monocytes. Readily detected transcripts include αENaC, βENaC, ASIC2, and ASIC3 in macrophage RNA samples, and αENaC and ASIC2 in freshly isolated bone marrow monocytes (Figure 1). Other ENaC and ASIC transcripts had very high C_Th_'s (βENaC, ASIC4/5), required more starting material (i.e., 1000 ng of RNA for ASIC1 vs. 100 ng for others), or inconsistent amplification within replicates (γENaC, ASIC1, and ASIC5). C_Th_ ranked lowest to highest: αENaC<ASIC3<βENaC≤ASIC2. With a few exceptions, we found a similar expression profile and C_Th_ ranking for ENaC and ASIC transcripts in freshly isolated monocytes using 75 ng RNA equivalent from monocytes pooled from *n* = 4 animals of each sex (Figure 1c). ASIC2b was not detected in any samples. Sex differences were identified for αENaC, γENaC, and ASIC4 expression in macrophages and γENaC in monocytes (Figure 1b,c). We confirmed expression of αENaC (~85 kDa) and βENaC (multiple bands ~75 and one ~250 kDa) by Western blotting (Figure 1d). We have observed higher than predicted molecular masses for βENaC in vascular smooth muscle cells (Grifoni et al., 2006; Lu et al., 2022).

### 3.2 Characterization of bone marrow derived macrophages

**FIGURE 2:**
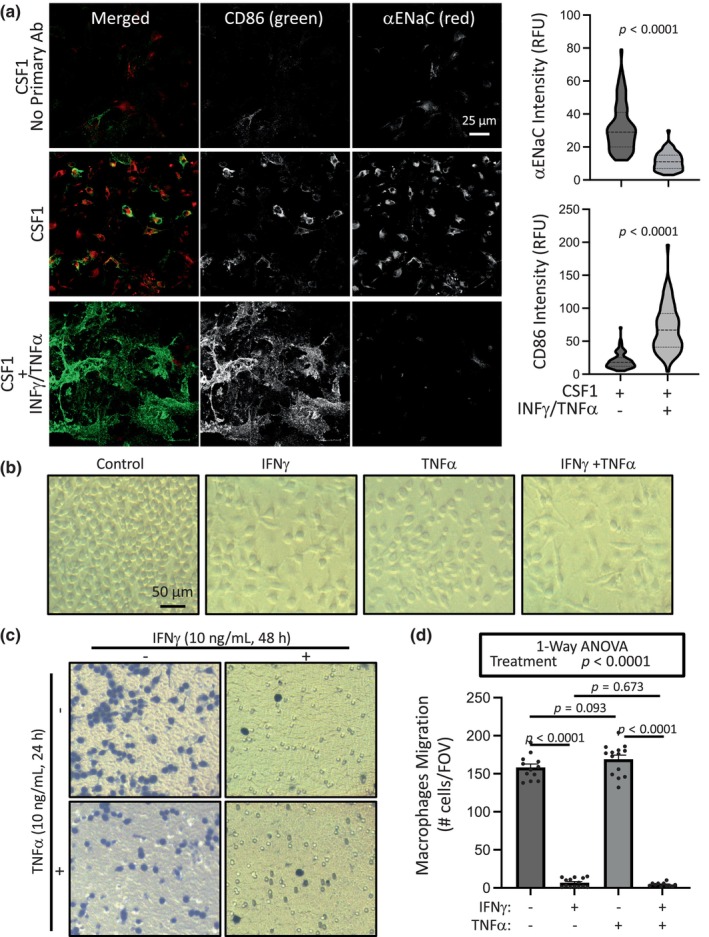
Proinflammatory cytokines IFNγ + TNFα on CD86/αENaC expression and chemotactic migration in bone marrow macrophages. Cells (female) were grown in the presence of CSF1 for 7 days. IFNγ (10 ng/mL) and TNFα (10 ng/mL) were added for the last 48 h and 24 h, respectively. (a) Representative images (left) and group data (right) showing αENaC expression is inhibited while CD86 expression is upregulated with polarization to M1 macrophages following IFNγ + TNFα treatment (*n* = 73–75 cells from *n* = 3 images per group). Data analyzed using a two‐tailed, Mann–Whitney test. (b) Light microscopy images of bone marrow macrophages treated with TNFα ± IFNγ prior to migration. Cells exhibit morphological changes consistent with polarization: Cells become flatter, less round and enlarged with processes. (c) Representative images of the underside of migration membranes where cells are stained blue. (d) Group data showing IFNγ, with or without TNFα, suppressed 24 h migration. TNFα had a negligible impact on migration. Data are mean ± SEM and analyzed using independent, two‐tailed *t*‐tests (a, b) or one‐way ANOVA, followed by Holm‐Sidak post hoc test (d). *p* values of differences among groups are shown on graph and demonstrate confidence.

We have previously examined the importance of proinflammatory cytokine (IFNγ and TNFα) regulation on macrophage morphology, migration and αENaC expression and wanted to determine if a similar relationship occurs in bone marrow derived macrophages (Nemeth et al., 2022). Similar to RAW cell line, expression of αENaC is abolished and CD86, a marker of activated macrophages (M1), is enhanced following exposure to bone marrow derived macrophages to IFNγ and TNFα. Representative images and quantitative data are provided in Figure 2a. Images in Figure 2b show polarization of bone marrow derived macrophages following treatment with IFNγ, TNFα, or both, characterized by cell loss, and enlargement and flattening of surviving cells. Exposure to IFNγ or IFNγ plus TNFα, but not TNFα alone, inhibited migration of bone marrow derived macrophages. Representative images of the underside of the migration membrane and quantitative data are shown (Figure 2c,d). These findings suggest that bone marrow derived macrophages generally behave similarly to IFNγ and TNFα in regards to αENaC expression, morphological changes, and migration responses.

### 3.3 Role of degenerins in migration of bone marrow derived macrophages

**FIGURE 3:**
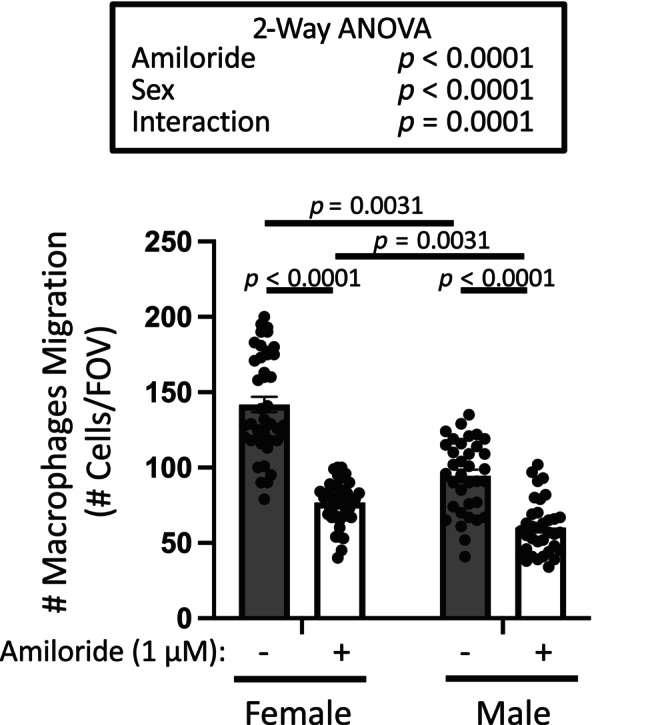
Broad spectrum degenerin inhibitor amiloride inhibits migration in male and female bone marrow derived macrophages. Degenerin inhibition with amiloride inhibited migration to 56% and 62% in female and male macrophages, respectively, of control. Baseline migration was greater in female macrophages. Macrophages from male and female animals were treated, migrated, and quantified side‐by‐side in two independent trials. Data are mean ± SEM and analyzed using two‐way ANOVA, followed by Holm‐Sidak post hoc test. *p* values of main factors and their interaction and differences among groups are shown on graph to demonstrate confidence.

**FIGURE 4:**
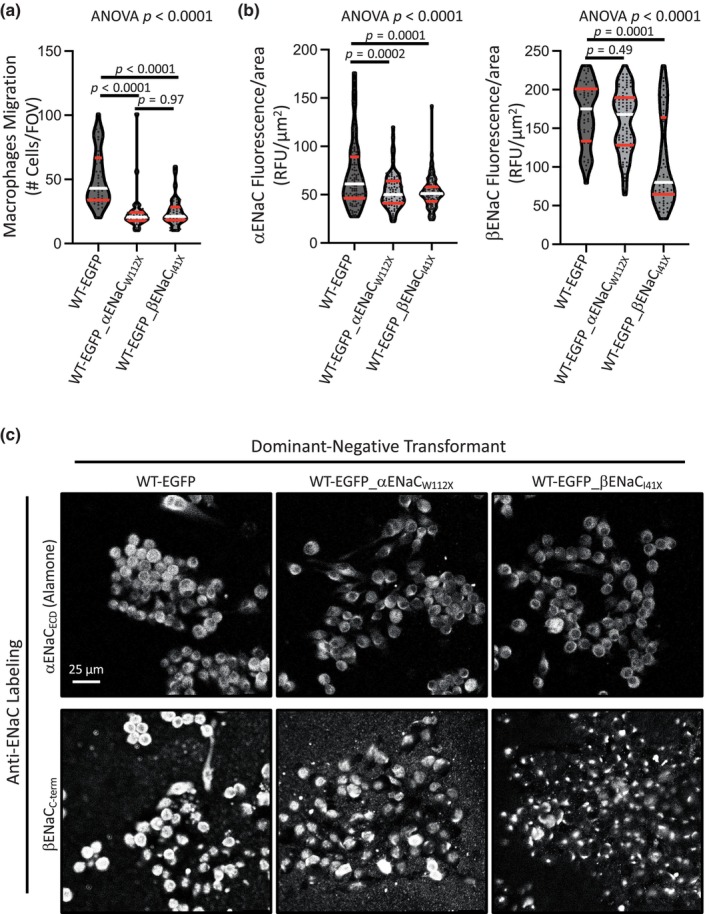
Silencing of αENaC and βENaC in bone marrow macrophages inhibits chemotactic migration. (a) Plots showing chemotactic migration responses in wildtype (WT) female cells stably transfected with EGFP_αENaCW112X or EGFP_βENaCI41X are suppressed greater than 50% of control (EGFP). Cells were maintained in the presence of G418 (1:100). Group data (b) and representative images (c) of semiquantitative immunolabeling shows αENaC and βENaC expression (Donkey anti‐Rabbit Alexa 546) is suppressed in transfected cells. Migration data represent seven FOVs, from three inserts, in two independent trials. Immunolabeling represent *n* = 50–100 cells from *n* = 2–4 images. Antibodies are directed to sequences downstream of the dominant‐negative construct. All data are mean ± SEM and analyzed using Brown Forsyth‐Welch Test followed by Dunnett post hoc test. *p* values are provided on graph and demonstrate confidence.

To determine the importance of degenerins in migration of bone marrow derived macrophages, we used 1 μM amiloride, a broad‐spectrum inhibitor. As shown in Figure 3, incubating cells with amiloride for 30 min prior to and during migration, reduced chemotactic migration to 56% and 62% of control in female and male macrophages, respectively. Macrophages from female mice showed a greater control migration capacity than macrophages from male mice. To determine the importance of αENaC and γENaC in macrophage migration, we used gene silencing in stably transfected cells from female mice. Silencing of αENaC or βENaC suppressed chemotactic migration by 52% and 51% of control, respectively (Figure 4a). The extent of αENaC and βENaC silencing using quantitative immunolabeling are shown in Figure 4b. Representative immunolabeling images are shown in Figure 4c. Amiloride and gene silencing had a similar impact on migration inhibition. These data support a role for degenerins, including αENaC and βENaC in bone marrow derived macrophage migration.

### 3.4 Role of select degenerins in freshly isolated bone marrow monocyte migration

**FIGURE 5:**
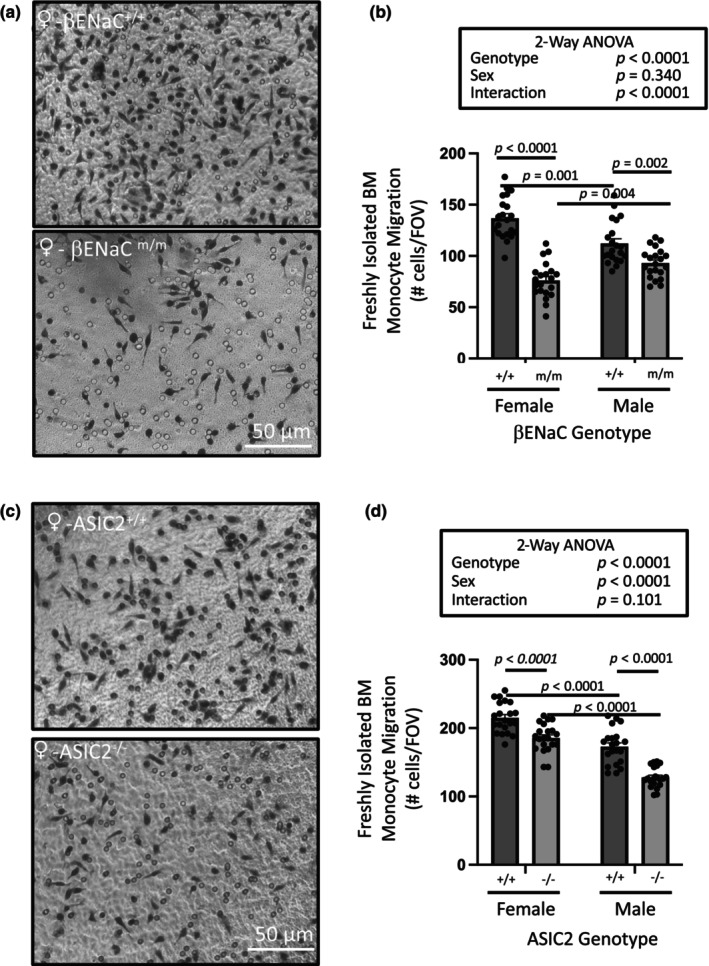
Loss of βENaC or ASIC2 inhibits bone marrow monocyte chemotactic migration. Chemotactic migration is attenuated in freshly isolated bone marrow monocytes from (a, b) βENaC hypomorph mice (βENaC^m/m^, 10–13 weeks of age) or (c, d) ASIC2 global knockout mice (ASIC2^−/−^, 7–8 weeks of age). Monocytes were isolated in parallel from an age‐matched wildtype and modified animal within a sex then migrated overnight. Mice were used between 7 and 13 weeks of age. These findings suggest (1) migration responses in wildtype mice are greater in female versus males, and (2) βENaC and ASIC2 both contribute to migration, but differentially in the sexes. Loss of βENaC had a larger impact on migration in female versus male, while loss of ASIC2 had a larger impact in males. Normalized migration data are shown in Figure Representative images are shown in panels a and c and group data are shown in panels b and d. Data are mean ± SEM and represent seven FOVs from three insets (*n* = 21) and were analyzed using two‐way ANOVA followed by Holm‐Sidak post hoc test. *p* values of main factors and their interaction and differences among groups are shown on the graph and demonstrate confidence.

**FIGURE 6:**
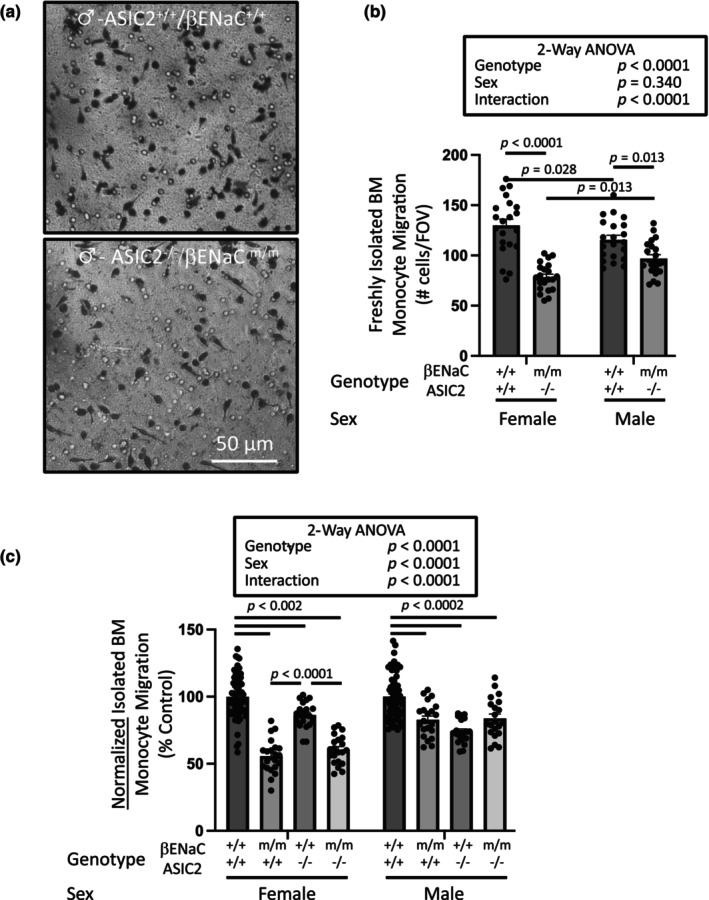
Loss of ASIC2 plus βENaC on bone marrow monocyte chemotactic migration is not additive. Chemotactic migration in freshly isolated bone marrow from mice homozygous for ASIC2 global knockout and βENaC hypomorph alleles (ASIC2^−/−^/βENaC^m/m^, 6–7 weeks). Monocytes were isolated in parallel from an age‐matched wildtype and modified animal within a sex then migrated overnight. Representative images of underside of migration membrane (a) and group data (b) in males are shown. These findings suggest (1) migration responses in wildtype mice are greater in female versus male, and (2) βENaC plus ASIC2 both contribute to migration, but greater impact on female. Loss of βENaC had a larger impact on migration in female versus male, while loss of ASIC2 had a larger impact in males. (c). Normalized migration data in monocytes from βENaC^m/m^, ASIC2^−/−^, and ASIC2^−/−^/βENaC^m/m^ mice are shown. Both data sets are presented as mean ± SEM and represent seven FOVs from three insets (*n* = 21, except wildtype control in C where *n* = 21 FOVs from *n* = 9 inserts) and were analyzed using 2‐way ANOVA followed by Holm‐Sidak post hoc test. *p* values of main factors and their interaction and differences among groups are shown on the graph and demonstrate confidence.

To determine if the migratory response in primary bone marrow derived monocytes is also dependent on select degenerins, we studied migration responses of monocytes from βENaC hypomorph (βENaC^m/m^) and global ASIC2 knockout (ASIC2^−/−^) mice as these models are available in our laboratory. Mice with global deficiencies in αENaC are not available as they die shortly after birth and γENaC was not detectable in female bone marrow monocytes. Representative images of the underside membrane (Figure 5a,c) and group data (Figure 5b,d) are shown for βENaC^m/m^ and ASIC2^−/−^ mice, respectively. Migration of bone marrow monocytes was suppressed in βENaC^m/m^ and ASIC2^−/−^ mice; however, sex and genotype dependent differences were evident. Similar to macrophages, monocytes from female mice had a greater migration response than monocytes from males. In female mice, migration was inhibited to a greater extent in βENaC^m/m^ than ASIC2^−/−^ mice. In male mice, migration was inhibited to a greater extent in ASIC2^−/−^ than βENaC^m/m^ mice. Migration responses in monocytes from mice carrying both mutations (ASIC2^−/−^ x βENaC^m/m^) were also attenuated; migration was inhibited to 60% of control in female versus 81% in male monocytes (Figure 6a,b). Comparison of normalized migration responses in freshly isolated bone marrow monocytes from the three genetic models is shown in Figure 6c and suggests loss of both subunits does not have additive effect on monocyte migration inhibition, a finding consistent with the possible formation of heteromeric channels containing at least βENaC and ASIC2.

### 3.5 Is there a link between migration and polarization status in bone marrow derived macrophages?

**FIGURE 7:**
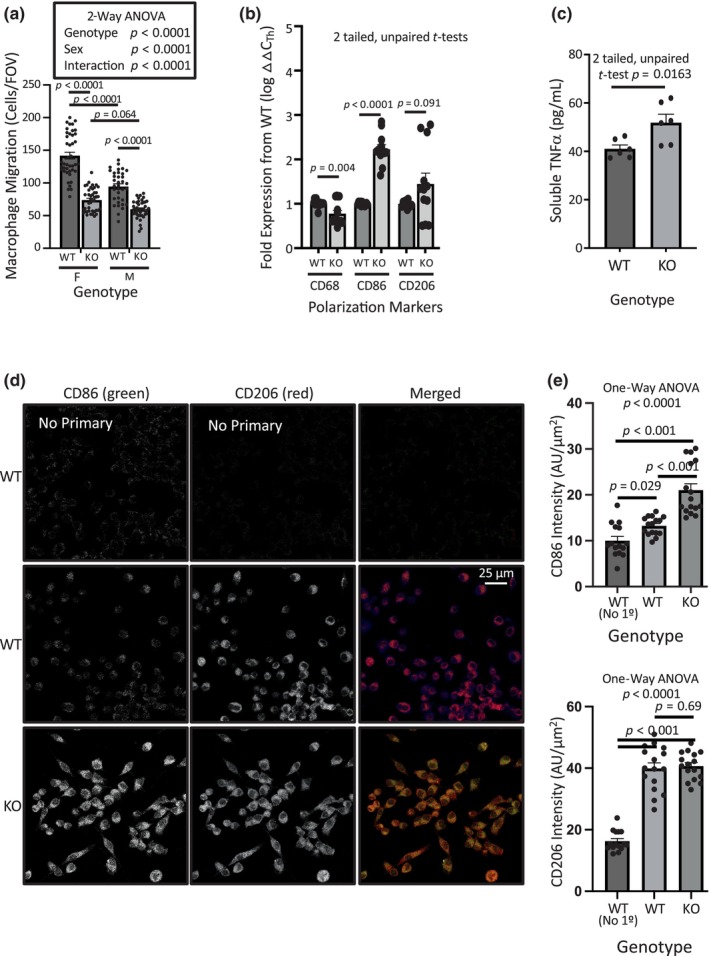
Bone marrow macrophages from mice lacking ASIC2 plus βENaC (ASIC2^−/−^/βENaC^m/m^, KO) are polarized towards an M1 phenotype. (a) Migration of bone marrow derived macrophages from ASIC2^−/−^/βENaC^m/m^ mice are inhibited to 55% and 65% of WT control cells from females and males, respectively. Data were analyzed using 2‐way ANOVA followed by Holm‐Sidak post hoc test. (b) Fold expression of monocyte/macrophage marker message in cultured bone marrow macrophages from males. The myeloid origin marker CD68 was decreased and M1 macrophage marker CD86 was upregulated in KO cells. The M2 marker CD206 was not significantly elevated. CD163, another marker of M2 macrophages, did not amplify in any samples. (c) Media soluble TNFα, released from M1 macrophages, was elevated in KO cell culture media. Samples were obtained from two wells from three different cell lines. Data in Panels b and d were analyzed using independent/unpaired, 2‐tailed *t*‐tests. Representative images (d) and group data (e) from semiquantitative immunolabeling of CD86 and CD206 in cells show CD86, but not CD206, are elevated in KO cells. each data point represents a cluster of cells, *n* = 5–6 cell clusters from *n* = 3 images. Fluorescence is normalized to cell area. Data in panels e and f analyzed using 1‐way ANOVA followed by the Holm‐Sidak post hoc test. These findings suggest bone marrow macrophages from mice lacking βENaC and ASIC2 are polarized towards the M1 phenotype. All data are mean ± SEM. P values are provided to demonstrate confidence.

Polarization of macrophages to a proinflammatory phenotype is associated with decreased macrophage mobility and upregulation of select expression markers (Cui et al., 2018; Vogel et al., 2014). Our previously published data in the monocyte–macrophage RAW cell line showed degenerin inhibition with amiloride increased expression of CD86, a proinflammatory marker. However, it was unclear if any degenerins were directly linked to migration and polarization status. To begin to address this issue, we first examined migration and phenotype marker expression in cultured bone marrow derived macrophages from control and βENaC^m/m^ x ASIC2^−/−^ mice. As expected, we found a loss of βENaC and ASIC2 suppressed chemotactic migration (Figure 7a). Consistent with a pro‐inflammatory phenotype, we found an upregulation of the inflammatory M1 marker CD86 message (Figure 7b). CD68 expression, a myeloid origin marker, decreased, and CD206, an anti‐inflammatory M2 marker was not statistically different. We were unable to detect expression of CD163 by qPCR, an additional M2 marker in control or treated cells (data not shown). Semiquantitative immunolabeling data support the upregulation of CD86 and unchanged CD206 expression as shown by the representative images and group data (Figure 7d,e). Secreted proinflammatory cytokine TNFα in the cell culture media was also increased (Figure 7c). We were unable to detect TGFβ, IL1b, IL6, IL10, IL17, or iNOS in cell culture media.

**FIGURE 8:**
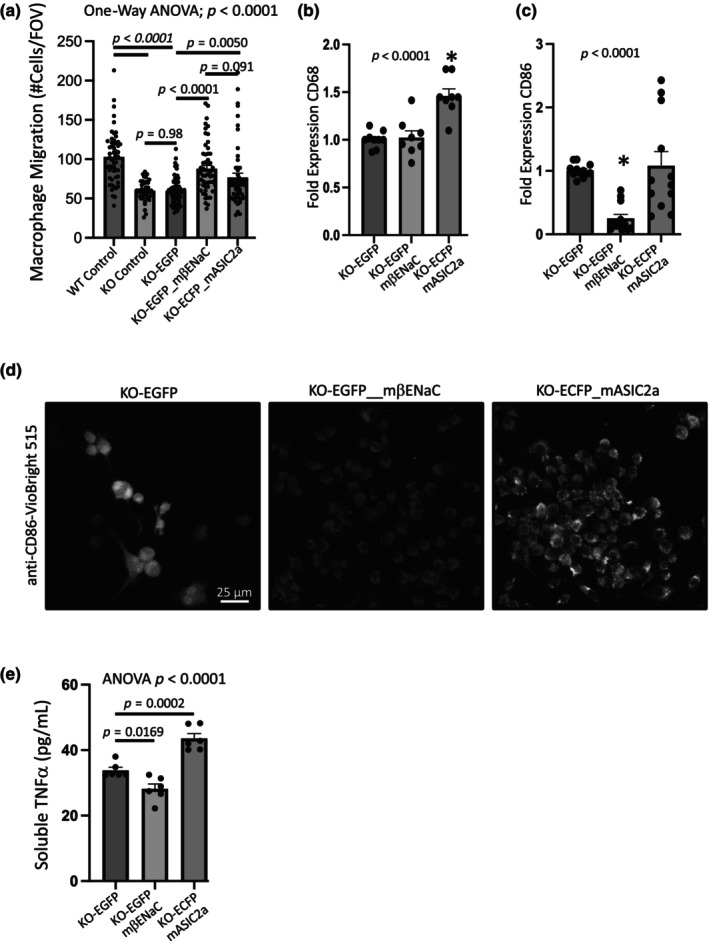
Does rescue of ASIC2 or βENaC in bone marrow macrophages from ASIC2^−/−^/βENaC^m/m^ mice restore the chemotactic migration response and polarization marker expression? ASIC2^−/−^/βENaC^m/m^ (KO) male cell lines were transfected with ECFP_mouse ASIC2 or EGFP_mouse βENaC full length constructs and maintained in the presence of selection antibiotic G418 (except WT control and KO control). (a) Rescue of either ASIC2 or βENaC partially rescues the chemotactic migration response in macrophages lacking ASIC2 and βENaC. Migration data points represent 42–63 FOVs from *n* = 2 to 3 inserts from three trials. (b) The monocyte origin marker CD68 (b) increased with rescue of ASIC2a and M1 macrophage marker CD86 decreased (c, d), consistent with the decrease in CD68 and increase in CD86 in KO versus WT macrophages (Figure 7). The C_Th_ for GAPDH, CD68, and CD86 in KO‐EGFP control samples are within range to detect increases or decreases (20, 20, and 32, respectively). CD163 were not consistently detected in the three replicates from 2 to 5 independent trials. (d). Immunolabeling of CD86‐Viobright 515 in KO macrophages rescued with βENaC or ASIC2 are consistent with qPCR findings. Fluorescence signal of CD86 was greater than baseline EGFP/ECFP signal assessed in separate samples (not shown). (e) Soluble TNFα in the media of cultured macrophages (72 h) was decreased in βENaC, but increased in ASIC2 rescued macrophages. All data are mean ± SEM. Migration data were analyzed using 1‐way ANOVA followed by Holm‐Sidak post hoc test. Quantitative PCR were analyzed using a 1‐way ANOVA followed by Dunnett post hoc test. *p* values are provided to demonstrate confidence.

Rescue of βENaC, and ASIC2 to a lesser extent, partially rescued the migration response (Figure 8a). Reintroduction of ASIC2 rescued CD68 expression (Figure 8b). Reintroduction of βENaC rescued CD86 expression as quantified by qPCR and immunolabeling (Figure 8c,d). Reintroduction of βENaC rescued the elevated soluble TNFα in the cell culture media (Figure 8e). Unexpectedly, reintroduction of ASIC2 elevated TNFα above KO‐EGFP control. Taken together, these findings suggest βENaC and ASIC2 differentially contribute to macrophage polarization status.

## 4 DISCUSSION

This investigation examined expression of the degenerin proteins, an evolutionarily conserved family of cation ion channels that function in epithelial Na^+^ absorption, extracellular proton sensors, neurotransmitter gated channels, and mechanosensors, in their role in bone marrow derived monocyte–macrophage polarization and migration (Bianchi & Driscoll, 2002; Kellenberger & Schild, 2002; Syntichaki & Tavernarakis, 2004). The major findings of this investigation demonstrate that most degenerins are expressed in bone marrow derived monocytes and macrophages and select degenerins contribute to chemotactic migration and polarization status. Interestingly, we found bone marrow derived monocytes and macrophages from female mice migrate to a greater extent than males. We also found that loss of at least ASIC2 and/or βENaC favors partial polarization towards a proinflammatory phenotype and rescuing expression recovers migratory capacity and phenotypic markers.

### 4.1 Bone marrow monocytes and derived macrophages have similar degenerin subunit expression pattern

Most degenerin subunits are expressed in bone marrow derived macrophages and freshly isolated monocytes, with minor sex differences. Degenerin message is expressed at relatively low levels with C_th_'s greater than 30 for all subunits examined. αENaC had the lowest C_th_, followed by βENaC, ASIC1, ASIC2, and ASIC3. γENaC, ASIC4, and ASIC5 had the highest C_th_'s. We also found αENaC is the most abundantly expressed degenerin message in monocytes and our previous findings in the RAW cell macrophage cell line (Nemeth et al., 2022). Our current findings suggest sex differences in macrophage expression of αENaC, γENaC, and ASIC4; C_th_'s for αENaC and ASIC4 were higher in females. In bone marrow monocytes, αENaC and ASIC2 were the most easily detected degenerin subunits and we were unable to detect γENaC in females. The physiological relevance of these sex differences is unclear.

### 4.2 Is the degenerin expression pattern consistent across monocyte derived cells?

Our findings in bone marrow monocytes and differentiated macrophages share similarities and differences in degenerin expression to peripheral blood mononuclear cells, bone marrow derived dendritic cells, splenic dendritic cells, and the monocyte–macrophage RAW cell line. Similarities include apparent robust expression of αENaC and weak or lack of expression of γENaC in PBMCs, RAW cells, splenic dendritic cells, and bone marrow monocytes and derived macrophages (Barbaro et al., 2017;Ertuglu et al., 2024; Nemeth et al., 2022). ASIC1‐3 are expressed in RAW cells, bone marrow derived dendritic cells, and bone marrow monocytes and derived macrophages (Ni et al., 2018; Tong et al., 2011). In contrast, PBMC monocytes do not express βENaC, ASIC1, or ASIC2 (Ertuglu et al., 2024). Additionally, splenic dendritic cells do not express βENaC (Barbaro et al., 2017). Several factors may account for these variations. First, macrophages have a heterogeneous origin, including bone marrow and tissue resident cells, and undergo different differentiation processes which may contribute to the phenotypic and gene expression differences (Naito, 1993; Zhao et al., 2018). Second, the hormonal and cytokine mileu of the preparation (i.e., blood from hypertensive patients, presence of supplemental growth/survival factors, and serum content in cell culture environment) likely influences expression of the degenerin expression pattern. The robust expression of serum glucocorticoid kinase 1, an important regulator of ENaC expression, in PBM monocytes and dendritic cells and the sensitivity of monocyte–macrophage degenerin expression to proinflammatory cytokines are two findings that are consistent with this possibility (Ertuglu et al., 2024; Nemeth et al., 2022).

### 4.3 Bone marrow derived macrophages have a similar response to proinflammatory cytokines as the monocyte–macrophage RAW cell line

When exposed to the proinflammatory cytokines IFNγ and TNFα, bone marrow derived differentiated macrophages exhibited morphological changes (enlargement, flattening, and reduced circularity), cell loss, reduced αENaC expression, and suppressed chemotactic migration similar to our previous findings in the RAW cell line (Nemeth et al., 2022). In contrast to the RAW cell line, bone marrow derived macrophages required a survival factor such as CSF1 or CSF2 to proliferate in culture.

### 4.4 Degenerins are required for normal bone marrow derived macrophage and monocyte chemotactic migration

We used several approaches to determine the importance of degenerins in migration. We initially used the broad spectrum degenerin inhibitor amiloride and found 1 μM inhibited migration to 56% and 62% in female and male macrophages, respectively, of control. The IC_50_ of amiloride for ENaC is ~0.1 μM and 10 μM for ASIC channels, the concentration of amiloride used in the current study, favors inhibition of ENaC channels and ASIC channels are blocked to a lesser extent (Lingueglia & Lazdunski, 2013). Chemotactic migration in macrophages was similarly inhibited using gene silencing approaches. We also examined the importance of select degenerins in macrophage migration using dominant negative directed gene silencing (αENaC and βENaC) and genetically modified (ASIC2^−/−^ × βENaC^m/m^) animals. We found chemotactic migration of macrophages were inhibited to a similar extent (~50%), regardless of the approach. Since degenerins are known to form heteromeric channels, namely, it is likely that some degenerins may associate to form multimeric channels in macrophages as well. This possibility is supported by our findings in freshly monocytes; while the loss of βENaC or ASIC2 alone had differing impacts on migration inhibition, they were not additive.

### 4.5 What is the role of other degenerins ASIC1 and ASIC3 in migration?

Although we detected expression of other ASIC channels in addition to ASIC2, we did not directly examine their role in migration. Other reports have addressed the role of ASIC1 using psalmotoxin, an inhibitor of ASIC1 homomeric, but not heteromeric, channels (Ni et al., 2018) on wound healing migration. The tendency for ASIC subunits to form heteromeric channels with no selective pharmacologic blocking or activating agents makes this challenging without gene silencing or knockout models. Extracellular proton gated ASIC‐like currents are present in dendritic cells; however, their molecular identity is undetermined (Tong et al., 2011). While ASIC1 and ASIC2 may be positive regulators of migration, other ASIC channels may not play a similar role. Not all ASIC subunits contribute similarly to vascular smooth muscle cell and glial migration (Grifoni et al., 2008; Kapoor et al., 2009; Vila‐Carriles et al., 2007).

### 4.6 Greater migration response in monocytes and macrophages from females compared to males

We consistently found that the chemotactic migration response in control animals were greater macrophages and monocytes from female versus male mice, a novel finding not previously reported to our knowledge. The mechanisms underlying this difference are unclear but may influenced by female sex steroids on ENaC expression as sexual dimorphism in ENaC expression in renal tubular tissue and endothelial cells (Gambling et al., 2004; Padilla et al., 2019; Veiras et al., 2017).

### 4.7 Why isn't there a more profound polarization following loss of ASIC2 and βENaC?

In our current and previous study using pharmacological ENaC inhibition, we found a modest polarization to the M1 phenotype (Nemeth et al., 2022). The current study provides direct evidence that βENaC and ASIC2 contribute to M1 polarization as rescue of either subunit differently contribute to normalization of CD86 expression and TNFα secretion in the macrophages lacking ASIC2 and βENaC. This finding, combined with our finding that polarization with IFNγ/TNFα suppresses at least αENaC expression (in RAW cells and bone marrow derived macrophages), suggests silencing of βENaC and ASIC2 is part of the proinflammatory cytokine‐initiated polarization signaling (Nemeth et al., Figure 2a,b). The impact of proinflammatory cytokines of expression on expression of other subunits have not been examined. The proinflammatory cytokines, including IFNγ, TNFα, and lipopolysaccharide, generally activate transcription factors (STAT1/3/6) which activate other signaling factors such as NOS or NFkβ (Murray, 2017; Orekhov et al., 2019). This raises the possibility that proinflammatory cytokine mediated inhibition of degenerins may be a mechanistic component of polarization patterning.

### 4.8 How do ENaC/ASIC channels regulate migration?

Mammalian degenerin channels are activated by multiple factors including neurohumoral (β2 adrenergic and mineral‐glucocorticoids), extracellular protons (ASIC channels), intracellular proteases (furin), and mechanical forces (shear stress and strain) (Fronius, 2013). αβγENaC channels are constitutively active channels that are sensitive to changes in extracellular Na^+^ (heterologous expression systems and renal collecting duct cells) and can be gated by shear stress (endothelial cells and renal collecting duct cells) and strain (vascular smooth muscle cells) (Awayda et al., 1995; Barbaro et al., 2017; Carattino et al., 2004; Jernigan & Drummond, 2006; Knoepp et al., 2020; Mano & Driscoll, 1999; Shi et al., 2013). Several ASIC channels are required for mechano‐dependent processes in touch receptors, baroreceptors, and VSMCs (Gannon et al., 2008, 2015; Lu et al., 2009, 2022; Price et al., 2000). Additionally, degenerin proteins have large extracellular domains (~300–400 amino acids) that form an antennae like structure that is likely to interact with the extracellular matrix (Welsh et al., 2002). Migrating cells are exposed to mechanical forces as monocytes “roll” along the intravascular space and as extracellular matrix contacts are disrupted then reformed to accommodate cell movement through tissue towards site of injury. Additionally, changes in cell volume and shape (forming leading and trailing edges) occur during migration. Thus, it is possible degenerins may participate in the signaling of mechanical forces may. We hypothesize that ENaC‐ASIC may play a role in sensing mechanical forces through interactions with the extracellular matrix and/or cell volume to contribute to signaling underlying migration.

## 5 SIGNIFICANCE AND PERSPECTIVES

Our findings suggest that transcripts for degenerin channels are expressed in bone marrow monocytes and macrophages and certain degenerins are required for monocyte/macrophage migration, a fundamental phenotype, which is dependent upon polarization status. Degenerins may act as sensors of the chemical and mechanical features of the extracellular environment to regulate M1 polarization and migration. How loss of degenerin function may affect phagocytosis of damaged tissue and polarization to the reparative M2 phenotype is unclear and will be determined by future studies.

## ETHICS STATEMENT

The authors have no conflicts of interest to declare.

## Data Availability

Data will be made available upon request.
